# Giant Lipoma of Transverse Colon Presenting With Partial Obstruction of Intestine

**DOI:** 10.7759/cureus.21651

**Published:** 2022-01-26

**Authors:** Asma Aqib, Asad Khan, Krishna Venkata

**Affiliations:** 1 Internal Medicine, University of Alabama, Montgomery, USA

**Keywords:** colonic lipoma, transverse colon, giant lipomas, intestinal obstruction, intestinal lipoma

## Abstract

Colonic lipomas (CLs) are a rare benign neoplasm of the gastrointestinal tract and the second most common tumor of the colon after adenomatous polyps. The clinical presentation of lipomas depends on the size of the tumor. Lesions that are less than 2 cm are mostly asymptomatic and are incidental findings during procedures like colonoscopies, imaging, or surgery. CLs that are greater than 2 cm can present with abdominal pain, nausea, intestinal obstruction, diarrhea, bleeding, and intussusception. Barium enema and colonoscopy can provide diagnostic clues; however, abdominal computed tomography (CT) confirms the diagnosis. Treatment modalities range from observation to segmental colectomy and endoscopic or laparoscopic procedures. Surgical resection is preferred for CLs that are either symptomatic or greater than 2 cm. Giant lipomas are often symptomatic and amenable to treatment, with surgical resection, laparoscopy, and endoscopic resection being the preferred treatment modalities. In this case report, we discuss a case of a 71-year-old white female who presented with abdominal pain and was found to have a relevant intestinal obstruction due to a 7.2 x 4.7 x 5.3 cm fatty mass arising from the transverse colon. Right hemicolectomy was performed, and histopathological examination revealed a lipoma of the transverse colon.

## Introduction

Intestinal lipomas are one of the rare types of gastrointestinal tumors [1,2]. The incidence of colonic tumors ranges from 0.035% to 4.4% in the autopsy series [3]. The peak incidence occurs within the fifth and sixth decades of life with a slight female predominance [4]. Colonic lipomas (CLs) are mostly solitary lesions. The most common locations are the ascending colon (45%), sigmoid colon (30.3%), descending colon (15.2%), transverse colon (9.1%), and rectum (3.4%) [2-5]. The CLs are mostly asymptomatic and do not require treatment; however, a small number of CLs (particularly those with a size >2 cm) require surgical management [6,7]. The term "giant lipoma" has been defined as a mass of >5 cm in diameter [8]. This case report encompasses a giant intestinal lipoma causing intestinal obstruction.

## Case presentation

**Figure 1:**
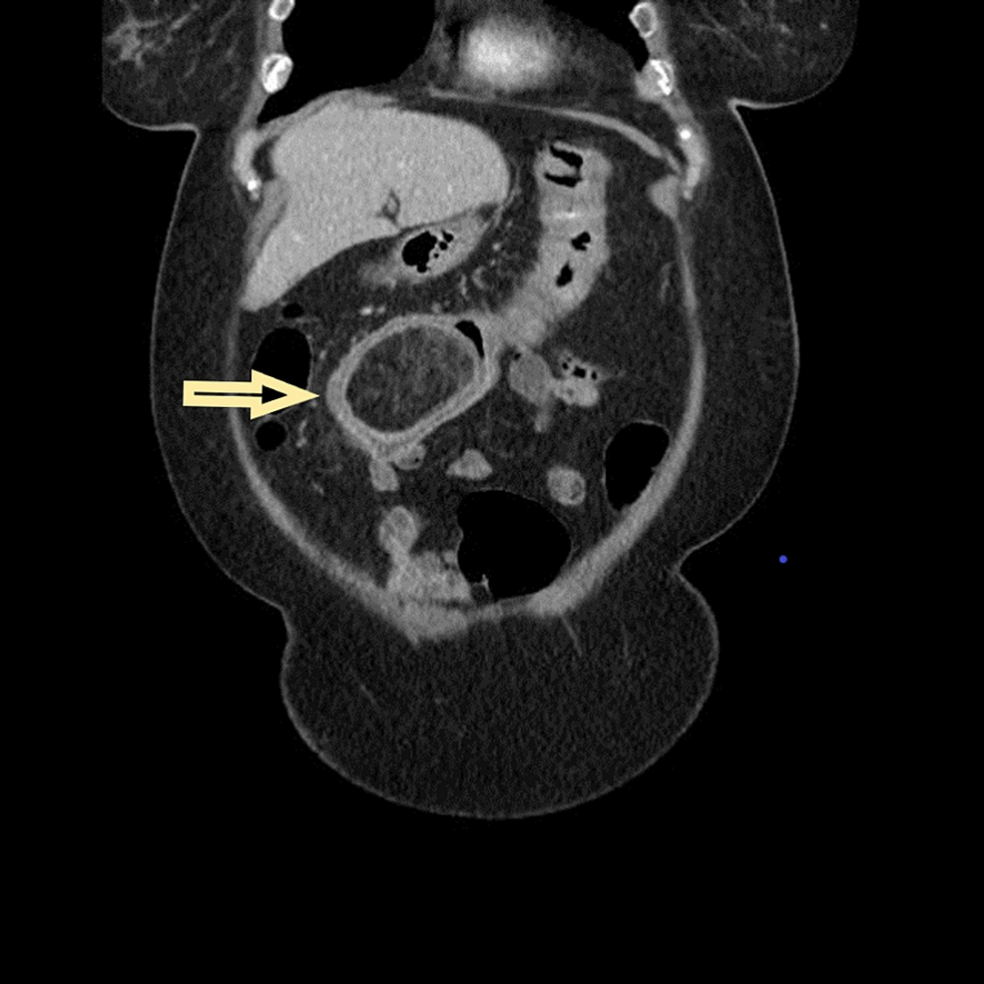
Preoperative computed tomography scan of the abdomen showing a 7.2 x 4.7 x 5.3 cm round mass of fat density representing lipoma within the lumen of the transverse colon. The yellow arrow indicates the giant colonic lipoma in the transverse colon.

A 71-year-old white female with no significant past medical history presented with a four-week history of moderate, intermittent, colicky upper abdominal pain and nausea. She denied vomiting, dyspepsia, diarrhea, melena, hematochezia, weight loss, prior esophagogastroduodenoscopy, or colonoscopy. On examination, she was afebrile and hemodynamically stable. Her abdomen was moderately tender at midepigastrium with hyperactive bowel sounds and no palpable abdominal mass. Rectal examination was unremarkable for masses or blood. The laboratory findings on admission were as follows: hemoglobin (11.6 mg/dl), white blood cell count (10,400/mm^3^), normal platelet count (251,000/mm^3^), potassium levels (3.1 mmol/L), alkaline phosphatase (136 units/L), and normal aspartate transaminase and alanine transaminase. The serum carcinoembryonic antigen, total protein, and serum creatinine levels were within the normal range. Abdominal CT of the bowel and mesentery demonstrated a remarkably abnormal transverse colon. There was a large fatty mass within the colon that measured 7.2 x 4.7 x 5.3 cm. The right colon was fluid filled suggestive of relative intestinal obstruction (Figure 1).

These findings suggested a large lipoma versus intussusception or a large lipoma causing intussusception. The colonoscopy revealed a large submucosal mass in the transverse colon. This mass was not amenable to a laparoscopic approach because of its size and surrounding inflammation. Open laparotomy surgical hemicolectomy was done. An intraoperative gross exam of the resected mass revealed a submucosal lipoma with significant overlying mucosal changes.

**Figure 2:**
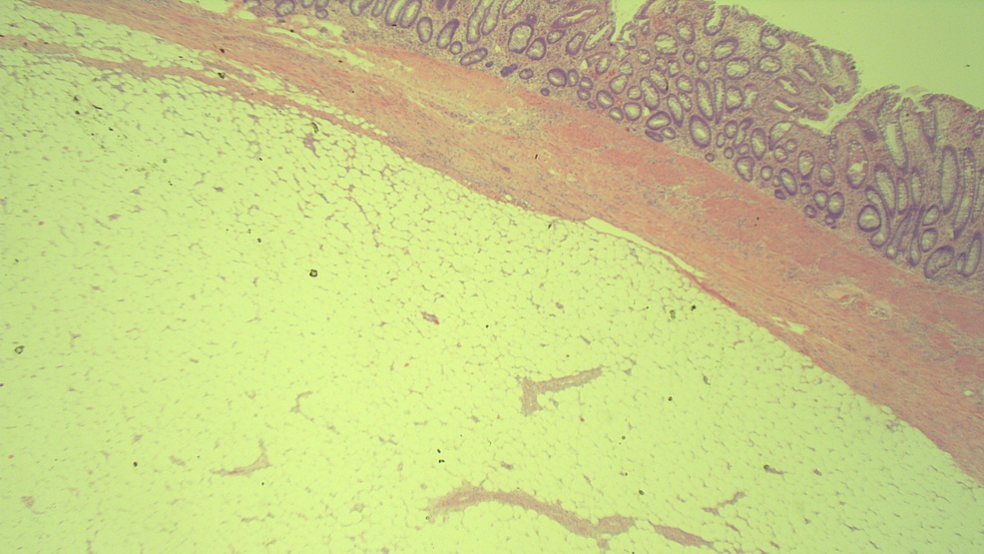
Histopathology of lipoma showing numerous fat cells.

Macroscopic pathological assessment of the resected specimen showed a bulbous mass within the central portion of the colon protruding into the lumen that measured 6.5 x 5.5 x 4.5 cm in greatest dimensions. There was superficial ulceration on this polypoid mass that had a uniform yellow lobulated fatty appearance throughout. The histological examination revealed colonic mucosa with ulceration, associated granulation tissue, and focal atypia. These findings suggested reactive changes without evidence of malignancy or adenomatous change (Figure 2).

The postoperative period is complicated by wound infection with *Escherichia coli* and *Enterococcus faecalis* secondary to anastomotic leak at the surgical site. The patient is treated with wound vacuum and antibiotics.

## Discussion

Intestinal lipomas smaller than 2 cm are mostly asymptomatic [9]. Approximately 25% of colonic lipomas are known to develop nonspecific symptoms like nausea, vomiting, and abdominal cramps once their diameter exceeds 2 cm [10]. The slow growth of the tumor can lead to progressive intestinal obstruction [9]. The most commonly reported symptoms are abdominal pain, constipation, diarrhea, bleeding, anemia, intussusception, and intestinal obstruction [5]. Colonic intussusception is one of the rare complications of colonic lipoma, reported in 1% of cases of intestinal obstruction [5,9].

Large colonic lipomas are often misdiagnosed as more serious pathology due to their rarity and variable presentation [2,11]. Lesions are mostly solitary, but multiple lesions have also been reported [12-15]. Ninety-five percent of the lesions are submucosal and may be sessile or pedunculated [2]. Pedunculated submucosal lipomas present with more significant clinical manifestations due to their great mobility [14]. Subserous lipomas are rather circular, do not present with mucosal abnormalities, and push back the colonic mucosa, which can lead to narrowing or blocking the colonic lumen [14].

These lipomas can introduce significant difficulty in generating a definite preoperative diagnosis. Three different examinations can provide arguments in favor of the diagnosis: barium enema, endoscopy, and endoscopic ultrasonography. Barium enema will show filling defects and a squeeze sign (change in luminal diameter during the peristalsis) [9]. Endoscopy may show a yellow-tinted polyp with thick pedunculated or broad-based attachment [2]. Endoscopic ultrasonography (EUS) can distinguish benign from malignant neoplasms and can determine the extent of tumor invasion before endoscopic resection [2].

A colonoscopy reveals various characteristics features of lipoma such as elevation of the mucosa stretched over the lipoma (“tenting sign”), an impression of soft mass under the biopsy forceps (“cushion sign”), and finally the visualization of yellow fat after performing a biopsy (“naked fat sign”) [16,17]. An abdominal CT scan is the most sensitive diagnostic test that shows fatty densitometric values of -40 to -120 Hounsfield unit (HU) with a smooth border and a uniform appearance [2,9]. Although MRI can be indicative of the diagnosis by showing fat suppressing images, further diagnostic confirmation is usually required. Therefore, CT is a commonly used diagnostic modality. Nonetheless, a definitive diagnosis is obtained by histopathological examination after resection. On gross examination, lipomas are soft, well-circumscribed, and compressible. On microscopic examination, these are well-differentiated adipocytes organized in lobules. Lipomas have no postoperative recurrence [9,18].

Treatment options vary from observation to endoscopic to laparoscopic or surgical removal [2]. Choice of treatment depends on the size, location, preoperative diagnosis, presentation, and morphology (sessile versus pedunculated) of the lipoma [2]. The endosonographic approach can be helpful for approachable lesions that are smaller than 2 cm. Treatment options include end loop excision, nylon loop assisted removal, endoclipping, and sectioning of overlying mucosa [2]. Laparoscopy and mini-laparotomy could be performed as an alternative to laparotomy or removal of lipomas as this reduces postoperative pain, disability, and recovery period [2,18]. Colectomy should be considered for lipomas larger than 2 cm when the diagnosis is obvious and complications are absent [2]. In all other cases, segmental colonic resection, hemicolectomy, or subtotal colectomy may be considered [2,18]. Some other operator-dependent endoscopic techniques mentioned in the literature include “Ligate and Let go” [18]. Endoscopic removal of lipomas has been recommended in certain situations [19].

## Conclusions

CLs are rare alimentary masses that can cause intestinal obstruction and intussusception. Colonoscopy and CT are useful diagnostic tools. The surgical approach is considered for lesions greater than 2 cm. Laparoscopy and mini-laparotomy could be future considerations for management. However, in some cases, the endoscopic reduction might be the optimal primary treatment modality. There is little to no chance of recurrence once CLs are removed.
